# Challenges of Hospital Payment Systems in Iran: Results from a Qualitative Study

**DOI:** 10.4314/ejhs.v34i3.8

**Published:** 2024-05

**Authors:** Shahriar Mokhtary, Ali Janati, Mahmood Yousefi, Behzad Raei, Fardin Moradi

**Affiliations:** 1 Department of Health Policy and Management, Iranian Center of Excellence in Health Management, School of Management and Medical Informatics, Tabriz University of Medical Sciences, Tabriz, Iran; 2 Health Economics Department, Iranian Center of Excellence in Health Management, School of Management and Medical Informatics, Tabriz University of Medical Sciences, Tabriz, Iran; 3 Department of Health Management and Economics, School of Public Health, Tehran University of Medical Sciences, Tehran, Iran

**Keywords:** Payment system, hospital, health care providers, qualitative study

## Abstract

**Background:**

The reform of hospital payment systems is a top priority for policymakers in many countries, including Iran. As knowledge of the current situation and experience with previous reforms are gained, the next phase will focus on improvement Therefore, this study aims to identify the challenges to hospital payments in the Iranian health system.

**Methods:**

This qualitative study used semi-structured interviews and focus group discussion meetings to collect data from 29 informants, including physicians, hospital administrators, faculty members, supervisors, and executive managers with expertise in hospital payment systems. Purposive sampling was used to recruit participants. Data were analyzed using content analysis.

**Results:**

The content analysis resulted in five themes and twenty-two sub-themes. Policy and regulation issues, payment methods, fair payment to providers, infrastructure and systems, and behavior of providers were cited as major challenges and drawbacks of Iran's hospital payment systems.

**Conclusions:**

Understanding the barriers to hospital payments is essential for reforming or alleviating the problem. This research has shed light on the current state of the hospital payment system in the Iranian health system. Knowledge of the issues with the current system and the needs of healthcare providers is essential for effective reform.

## Introduction

Hospitals in Iran play a significant role in providing high-quality healthcare services, with their workforce being essential to this mission (1, 2). One of the most critical control mechanisms for implementing health system reforms and achieving policymakers' objectives is the use of hospital payment systems (3). This reimbursement system is essential for the effectiveness of the healthcare system, which is one of the important responsibilities of the federal and state governments (4). One of the key elements in the healthcare sector is the capacity to secure adequate financial means to deliver healthcare services (5, 6). Governments worldwide, regardless of their income levels, express considerable apprehension regarding the augmentation of resources and effective allocation of health system resources (7). Implementing a well-structured payment system is crucial to curbing resource squandering and unnecessary patient interventions, as well as ensuring that healthcare providers receive adequate remuneration to motivate the provision of superior services (8, 9).

Various payment systems are implemented in different countries' healthcare systems according to their particular requirements and capacities (10). Iran, as a developing nation in the eastern Mediterranean region with a population of approximately 90 million people, has five main payment systems for its service providers: salary, capitation, fee-for-service, case-based payment, and performance-based systems (11). Family physicians who offer preventive care receive compensation through a salary system. Conversely, service providers in office and clinic settings mainly receive payment through a fee-for-service reimbursement system, though their compensation may also include salary components depending on the ownership structure. Apart from 90 specific procedures covered by a global budget, hospitals in the inpatient sector are remunerated using a fee-for-service reimbursement model (12).

Identifying and addressing challenges in these systems is crucial for enhancing healthcare quality, reducing informal payments, ensuring financial stability, and promoting equity. By understanding these challenges, policymakers can devise evidence-based solutions to enhance payment systems, encourage quality care, and combat issues such as delayed payments, inadequate reimbursement rates, and corruption. This study aims to explore challenges within Iran's hospital payment systems.

## Material and Method

**Study design and population**: This qualitative study (conducted from November 2021 to May 2022) used purposive sampling to select senior managers from Iranian healthcare and insurance sectors based on specific criteria. A mixed-methods approach, including interviews and focus group discussions (FGDs), was employed, with the triangulation technique for a comprehensive understanding. Judgment sampling for FGDs and snowball sampling for interviews were used to gather diverse perspectives. Integrating these methods provided complementary viewpoints on hospital payment system challenges (13).

**Focus group discussion**: Two FGDs with 16 participants were conducted using prompts to elicit rich data. Participants collaboratively scheduled 90-minute sessions, maintaining high group interaction through an associational context. Interaction patterns were analyzed to assess individual participation within the group setting (Table 1).

**Table 1:** The characteristics of the participants

Work experience (years)	Frequency	Position	Level	Type of organization
21	3	General manager of insurance	Top manager	Insurance
13	5	Budget and tariff managers	Middle manager	Ministry of health
17	3	Nursing manager	Top manager	University
9	7	Faculty member	Associate Professor	University
16	6	Physician	Specialist	Hospital
14	5	Administrative and support	Middle manager	Hospital

**Semi-structured interview**: Individual semi-structured interviews were conducted with participants unable to attend FGDs to gather in-depth personal experiences and thoughts on a specific payment method. An interview protocol with open-ended questions was tested for effectiveness. After obtaining consent, interviews were recorded, transcribed verbatim, and analyzed thematically through systematic coding. Tailored brief questions were used consistently by a skilled interviewer in 30-60 minute sessions until data saturation, totaling 13 interviews.

**Analysis and interpretation**: Content analysis was employed to extract concepts from FGDs and interview transcripts using an inductive approach (14). Two researchers were involved in the analysis process. They revisited interviews, identified questions, and created content summaries per participant. Themes and subthemes were indexed from the transcribed text. The researchers evaluated data for credibility, dependability, confirmability, cross-checking themes with participants, and maintaining comprehensive documentation (15).

Ethical guidelines were followed, including obtaining informed consent, ensuring participant anonymity and confidentiality, and obtaining ethical approval from Tabriz University of Medical Science (IR.TBZMED.REC.1401.358).

## Results

The characteristics of the participants are presented in Table 1.

The analysis of the interviewees revealed five themes and 22 subthemes regarding the weaknesses of hospital payment systems in Iran (Table 2). The main themes identified during the interview analysis included: 1) Policy and regulation, 2) Payment method, 3) Fair payment to providers, 4) Infrastructure and systems, and 5) Behavior of providers.

**Table 2:** The themes and subthemes of weaknesses of hospital payment systems

Themes	Subthemes
**Policy and regulation**	Unrealistic tariff rate
	Dual practice of physicians and nurses
	Weak clinical guidelines and prescribing recommendations
	Lack of transparent measures for payments and potential for informal payments
	Lack of adherence to risk- sharing
**Payment method**	Higher out of pocket payments
	Barriers to money streams from payers to providers
	Direct financial relationship between patients and physicians
**Fair payment to providers**	Gap in payments between the public and private sector and various health professionals
	Lack of Performance for pay
	Selective nature of patients (Cream skimming)
**Infrastructure and systems**	Financial and budgetary constraints
	Multiplicity of insurance fund financing
	Weakness of the referral system
	Weakness of the registration system and information structures
	Unbalanced geographic distribution
	Expensive monitoring and control methods by third-party payers
	Poor supervision of physician performance
**Behavior of providers**	Lack of incentives for doctors to work full-time in the public sector
	Decreased motivation to supply more services due to the gradual performance payment
	Induced demand and moral hazard
	Lack of incentive and motivation for efficient use of resources

### First theme: Policy and regulation

Six subthemes were extracted from this theme:

**Unrealistic tariff rate**: The issue of informal payments, as perceived by both healthcare providers and recipients, is often attributed to erroneous or impractical valuation and tariffing of services in both the private and public sectors.

*“Many of the fee-for-service (FFS) problems are related to unprincipled tariff setting, and by modifying the tariffs, the balance between different specialties and between the technical and the professional component can be improved.” (P3)*

**Dual practice of physicians and nurses**: The prevalence of dual practice among physicians, defined as working simultaneously in both the public and private sectors, is a widespread phenomenon in Iran. The concurrent involvement of professionals in both sectors can lead to conflicts of interest within the healthcare system.

*“Professionals, as brokers, in the public sector at the expense of the employer, which is the public hospitals, pursue the goals in the private sector, which is a manifestation of the conflict of interests in the health system.” (P1 and P 9)*

**Weak clinical guidelines and prescribing recommendations**: Participants in the study identified shortcomings in the process of defining and compiling clinical guidelines as one of the challenges faced by the payment system.

*“Deficiencies of clinical guidelines, training, supervision and some financial issues are effective in creating induced demand and unnecessary services.” (P22 and P25)*

**Lack of transparent measures for payments and potential for informal payments**: Iran's healthcare system is marked by significant out-of-pocket and informal payments. Study participants noted that the lack of transparency has enabled informal payments and occasionally resulted in the neglect of patients with serious illnesses.

*“More objective measures should be designed in payment. Lack of payment standards, performance and overtime payment as a matter of taste, the non-scientific basis of efficient calculation, are some of the serious challenges of the payments to hospital.” (P19 and P1)*

**Lack of adherence to risk-sharing**: Interviews revealed that the Iranian healthcare payment system does not adequately account for risk sharing, in that there is no proportionality between treatment outcomes and payments.

*“There is no linkage between the patient outcome and the amount of payment, and the payment system for hospitals is not such that there is a financial risk towards the service provider.” (P8)*

### Second Theme: Payment methods

This theme includes three subthemes:

**High out-of-pocket (out-of-pocket) payments**: Many respondents highlighted that Iran's elevated OOP payment rates adversely affect healthcare utilization and access for individuals.

*“In our country, 40-50% of the expenses are paid by the people, most of which are paid directly when receiving the service.” (P2)*

**Barriers to money streams from payers to providers**: In the Iranian health system, numerous obstacles hinder the transfer of funds from healthcare service payers to providers, potentially impeding financial stability in the system. Participants noted the following observations:

*“One of the challenges that hospitals are facing is the failure to pay money on time by insurance organizations. Insurance organizations pay the claims very late.” (P6)*

**Informal payments**: In Iran, the widespread prevalence of informal cash payments for healthcare services creates ambiguity in the financial relationship between physicians and patients.

*“If the income is too low, doctors will turn to under-the-table and illegal payments. The low salary level can mean that the efficient payment system has unofficially replaced the salary payment system” (P15)*

### Third theme: Fair payment to providers

In this theme, five subthemes were extracted as below:

**Gap in payments between the public and private sector and various health professionals**: In Iran, a significant income gap is seen between specialist physicians and nursing groups exists and professionals working in public and private sectors.

*“The average salary is not able to create welfare, which is caused by the inappropriate levels of payments between different groups, the disproportion between salaries and inflation, and the imbalance between the economy and the purchase price.” (P13 and P16)*

**Lack of performance for pay**: In Iran's healthcare system, there is a lack of correlation between performance and compensation.

*“Performance effectiveness and skill variables have little effect on the amount received. Note that even investing to improve productivity in performance-based payments may create costs for the organization that exceed the efficiency gains.” (P19)*

**Selective nature of patients (cream skimming)**: Cream skimming can significantly affect healthcare accessibility. Participants hinted at patient selection considerations happening in public hospitals.

*“Government hospitals provide a kind of subsidized care. Since the capacity of hospitals is fixed, such hospitals try to increase their profit margins by treating low-cost patients and visiting more patients.” (P14)*

### Fourth theme: Infrastructure and systems

This theme includes nine subthemes:

**Financial and budgetary constraints**: The interviewees enumerated the weaknesses of the financial and budget system and said that resources are not allocated sufficiently. They stated that there are limitations in financing that affect the payment system and wages.

*“Inadequate financial resources have also affected the payment system …” (P10)*

**Multiplicity of insurance fund financing**: The health insurance industry in Iran grapples with significant challenges arising from multiple decentralized insurance funds and fragmented decision-making on organization financing. Ineffective health financing mechanisms and overlapping coverage worsen these issues.

*“The health insurance organization and the social security organization, as two large insurance organizations, are financed from completely different sources, and their position is separate, which makes the issue of fund pooling challenging.” (P18)*

**Weakness of the referral system**: In the interviews, participants underscored concerns regarding patient movement within the referral system. Patients avoiding the system can more easily access specialist care directly.

*“The lack of financial relation between the doctor and the patient in the family medicine program and the lack of timely payment of salaries along with the budgetary pressure reduces the motivation to encourage patients to comply with the referral system.” (P21)*

***Weakness of the registration system and information structures:*** A major challenge in Iran's healthcare system is the deficiencies in the registration system and information structures. Study participants observed that the financial and payment information frameworks are susceptible to errors and biases, especially in registration and reporting.

*“One of the problems with the performance-based payment plan (Qasedak) in hospitals of Iran is the difficulty in recording basic information. Because this important basic information was manually transferred to this software, with a possibility of accidental or intentional mistakes.” (P19)*

**Unbalanced geographic distribution**: In Iran, the uneven distribution of health resources across geographic regions, especially the availability of general practitioners, highlights significant disparities.

*“Unbalanced distribution of inpatient and hospital treatment facilities in the country, while aggravating problems and shortages, has caused an imbalance in the distribution and employment of doctors.” (P27)*

***Expensive monitoring and control methods by third-party payers:*** Most participants in this study reported insurance monitoring and supervising the payment system in Iran is expensive and pluralistic.

*“Currently, there are people in the country who have been covered by several insurance funds; identifying these people is possible by collecting the insurances, and their overlapping is resolved and managed.” (P28)*

**Poor supervision of physician performance**: Inadequate oversight of physician performance presents a significant challenge within Iran's healthcare system. Participants in the study highlighted the incomplete and weak nature of physician performance monitoring.

*“Payment is based on taste, relations, and lack of supervision. Of course, each of these methods has its disadvantages and requires its own monitoring methods.” (P8)*

### Fifth theme: Behavior of providers

Seven subthemes were extracted from this theme:

**Lack of incentives for doctors to work full-time in the public sector**: Fee-for-service payment models across public and private sectors have caused health service providers to choose not to work full-time in the public sector. Instead, they opt to dedicate some or all of their time to working in the private sector.

*“The weakness of the policy of recruiting full-time doctors in public hospitals and the lack of competition between public and private hospitals have caused doctors to withdraw from full-time employment.” (P24 and P25)*

**Decreased motivation to supply more services**: The participants repeatedly spoke about the fact that in some cases, due to the low tariffs, doctors do not have the motivation to provide more services; this issue is more objective in performing surgeries.

*“With low tariffs on surgical services, there is no incentive to perform surgery - fee-based revenues do not meet the welfare needs of doctors.” (P14)*

***Induced demand and moral hazard:*** The payment system in Iran has incentivized some doctors to create unnecessary demand by taking advantage of unrealistic tariffs. Conversely, some individuals tend to overuse healthcare services due to the low tariffs and insurance premiums in the public sector.

*“The feeling of unrealistic tariffs makes the service providers refrain from contracting with the insurance organizations or causes the consumers to consume these services excessively (induced demand).” (P3)*

**Lack of incentive and motivation for efficient use of resources**: In Iran's healthcare system, there is a lack of incentives to efficiently use resources. This deficiency arises from weaknesses in the payment systems used in primary healthcare (PHC), where salaries and fee-for-service payments are prevalent.

*“Considering that the dominant hospital payment systems in Iran are based on salary and FFS, these payment methods do not create any direct motivation to try to manage resources and costs or to prescribe the most cost-effective healthcare interventions.” (P6)*

## Discussion

This study was conducted to pinpoint the challenges encountered by Iran's hospital payment system. Our discoveries underscore various critical challenges for policy development and regulation, such as unrealistic tariff rates, healthcare professionals engaging in dual practices, deficiencies in establishing clinical guidelines and prescribing protocols, absence of transparent payment metrics and susceptibility to informal payments, noncompliance with risk-sharing mechanisms, the considerable authority of physicians in decisionmaking, and the managerial structure of payments.

A study conducted by Barouni (2020) and our research both highlight significant challenges in policy, cost, regulation, and operation within the present payment system (16). Interviewees stressed the importance of combatting dual practice among physicians, which adversely affects patients by increasing out-of-pocket expenses and limiting healthcare accessibility, hindering the attainment of universal health coverage (17, 18). Concerns surrounding the establishment of medical tariffs in the Iranian healthcare system are prevalent, with scholars proposing that the direct financial dealings between physicians and patients, combined with insufficient physician income because of unrealistic tariffs, foster the acceptance of illicit payments (19, 20).

One critical payment concern in healthcare pertains to the deficiency in formulating clinical guidelines and prescribing recommendations. Current research suggests a scarcity of developed and effectively implemented clinical guidelines in Iran, encountering numerous barriers to distribution and utilization (21). The significance of clinical guidelines lies in maintaining an equilibrium between overuse and underuse of healthcare services (22). The inadequate uptake of clinical guidelines has been recognized as a factor exacerbating challenges in the payment system (23).

An additional issue in hospital payment systems involves the absence of clear payment measures and the potential for under-the-table payments. Research on Iranian payment systems highlights the lack of transparency and consistent payment methods in healthcare (24). Physicians in private healthcare settings often establish higher service fees, creating a potential conflict of interest as they have policymaking authority. Ebrahimi et al. discovered that around 20% of physicians receive financial incentives for directing patients to private clinics (25). Furthermore, Ghoddoosi-Nejad et al. stress the conflict of interest between the Ministry of Health and insurers as a significant hurdle in the strategic purchasing of healthcare in Iran (26).

The challenge of increased OOP payments and the direct financial connection between patients and healthcare providers is a notable concern. OOP payments act as obstacles to healthcare access and can result in severe financial strain for patients, leading to difficulties in obtaining services (27). Despite insurance coverage, patients are often inadequately shielded against medical costs, and OOP payments persistently escalate, even with insurance coverage. Moreover, insurance coverage tends to encourage households to utilize more healthcare resources by lowering healthcare costs (28).

Equitable payment is crucial for boosting job fulfillment, drive, and the quality of patient care. Mazdaki et al. observed a rise in medical specialists' earnings following the adoption of Iran's updated fee schedule (29). Leigh et al.'s research comparing 41 specialties noted that high-earning specialists predominantly concentrate on surgeries, advanced technologies, and expensive medications, while lower-paid specialists primarily focus on nonprocedural duties like patient interactions and examinations (30).

The lack of alignment between performance and payment poses a significant challenge in payment systems. Kondo et al. emphasize the necessity of establishing incentivizing measures and tying payments to these criteria for the effective implementation of a functional payment framework (31). In Iran's healthcare system, hurdles to implementing performance-based payment stem from issues related to behavior, organization, regulations, and rules, as noted by Hadian et al. (32). Najibi et al.'s study highlights those charitable hospitals in the Fars province encounter limitations such as diminished financial and human resources (33).

The division of the Iranian health insurance system has led to several challenges including insufficient financial security for insured individuals, fee-for-service payment structures for healthcare providers, and a scarcity of robust evidence for health insurance policy development (34). Bazyar et al. (2020) explored the consolidation of health insurance funds in Iran, emphasizing that fund pooling aids in supervising healthcare service provision levels. This consolidated approach also enables hospitals to partner with a single insurance body, thereby easing contract management, speeding up review processes, and guaranteeing effective reimbursement throughout the healthcare sector (35).

These findings have two main limitations. Firstly, the use of purposive sampling may introduce researcher bias and limit representation by omitting some viewpoints and experts. Secondly, the absence of a theoretical framework for data categorization and theme elicitation indicates a need for comprehensive analysis methods.

This research examined shortcomings and obstacles in Iran's current hospital payment system, spotlighting issues like inadequate reimbursement rates, lack of transparency, fragmented systems, and delays in payments. To enhance efficiency and transparency, reforms should be prioritized to streamline payment processes, guarantee proper reimbursement rates, enhance transparency, and encourage digitalization. Collaboration among stakeholders is essential for establishing a more effective and sustainable hospital payment system. The choice of payment mechanism should align with a country's context, management capabilities, and financial resources, as each method comes with its advantages and drawbacks. Complex payment methods may escalate administrative costs for healthcare organizations, emphasizing the absence of an ideal payment approach universally applicable across all healthcare entities.
